# Structures and photocatalytic properties of two new Zn(ii) coordination polymers based on semi-rigid V-shaped multicarboxylate ligands†

**DOI:** 10.1039/d0ra02222e

**Published:** 2020-05-18

**Authors:** Shan-He Zhou, Jun Wang, Yi-Wei Liu, Yuyu Zhong, Yan-Chun Sun, Bin Xie, Aiqing Ma, Amita Singh, Mohd. Muddassir, Abhinav Kumar

**Affiliations:** School of Chemistry and Environmental Engineering, Sichuan University of Science & Engineering Zigong PR China scwangjun2011@126.com; Dongguan Key Laboratory of Drug Design and Formulation Technology, School of Pharmacy, Guangdong Medical University Dongguan 523808 China maqandght@126.com; Department of Chemistry, Faculty of Science, University of Lucknow Lucknow 226007 India abhinavmarshal@gmail.com; Department of Chemistry, College of Sciences, King Saud University Riyadh 11451 Saudi Arabia

## Abstract

Two new metal–organic coordination polymers (CPs), aqua-2,2′-bipyridine-5-(4′-carboxylphenoxy)isophthalatezinc(ii) polymer [Zn(HL)(2,2′-bipy)(H_2_O)]_*n*_ (1) and tris-4,4′-bipyridine-bis-5-(4′-carboxylphenoxy)isophthalatetrizinc(ii) polymer [Zn_3_(L)2(4,4′-bipy)_3_]_*n*_ (2) (H_3_L = 5-(4′-carboxylphenoxy)isophthalic acid, 4,4′-bipy = 4,4′-bipyridine and 2,2′-bipy = 2,2′-bipyridine), were obtained under hydrothermal conditions and characterized by microanalysis, FTIR spectroscopy and single crystal X-ray diffraction. The single crystal X-ray diffraction indicated that in both the CPs the coordination networks exhibited varied topologies and coordination modes around the Zn(ii) centers. CP 1 exhibits a one-dimensional (1D) chain structure, which further forms a 3D supramolecular architecture *via* intermolecular π⋯π and hydrogen bonding interactions, while 2 possesses a 3D framework generated from a 2D layered motif comprising zinc and tripodal carboxylate subunits pillared by 4,4′-bpy ligands. Apart from the structural investigation, the photocatalytic performances of both the coordination polymers to photodecompose an aqueous solution of methyl violet (MV) were examined. The results indicated that both the CPs displayed the potential to photodecompose aromatic dyes and in particular 2 showed good photocatalytic activity for dye degradation under light irradiation. The photocatalytic mechanism through which these CPs executed degradation of dyes has been explained with the assistance of band gap calculations using density of states (DOS) and its decomposed partial DOS calculations.

## Abstract 2

Two new Zn(ii) coordination polymers having semi-rigid V-shaped polycarboxylate ligands were synthesized and their photocatalytic performances to photodegrade methyl violet were assessed.

## Graphical abstract

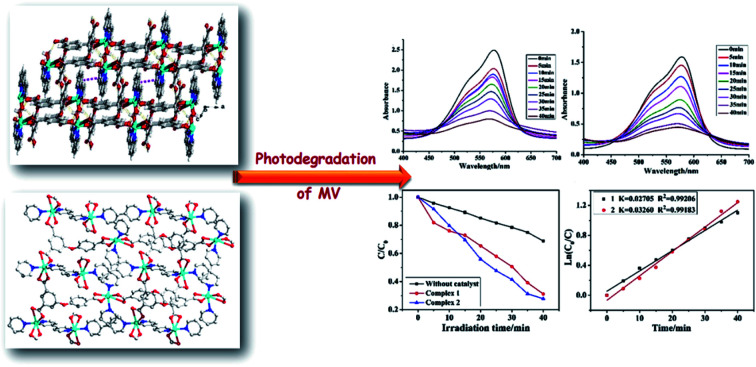

## Introduction

During last couple of years, research dealing with the development of new photocatalysts for the effective degradation of aromatic dyes – lethal contaminants existing in the wastewater discharge – has gained utmost importance.^1–3^ This is because photodegradation is believed to be one of the most promising strategies used for removal of water contamination.^1–3^ This technique can now be considered to be a green ecological technique as it decomposes organic aromatic compounds in waste-water discharge without adding any contaminants to the water, hence forming a basis for sustainable methodology.^1–3^ Although to date a variety of photocatalytic materials have been developed, recently, CP/MOF based photocatalysts have been used in the degradation of environmental pollutants.^4,5^ The CPs/MOFs not only find applications as photocatalysts but they also offer application as catalysts in hydrogen evolution and carbon dioxide reduction.^4,5^ In one of the pioneering works, the application of MOF-5 was explored for the photocatalytic degradation of phenol in aqueous medium.^6^ Also, Zn_3_(BTC)_2_ film had been studied for the degradation of methylene blue (MB).^7^ The most trustworthy method for the syntheses of the targeted CPs that can find application as photocatalysts is the mixed-ligand synthetic strategy.^8^ In this approach, typical building blocks are bipyridine (which can exist in three positional isomeric forms *viz.* 2,2′-; 3,3′- and 4,4′-) and polycarboxylate linkers. These are sometimes termed as acid–base mixed-ligand systems.^8^ In comparison to the rigid aromatic polycarboxylate ligands having one phenyl ring as the central molecular framework, the so-called semi-rigid V-shaped multicarboxylate ligands with two phenyl rings bridged by a CH_2_ or O entity as central molecular framework increases the flexibility of the ligand. These semi-rigid V-shaped multicarboxylate ligands can yield new metal complexes with diversified structures and topological features because of the free rotation of two aromatic rings around the bridged CH_2_ or O entity.^9,10^

**Scheme 1:**
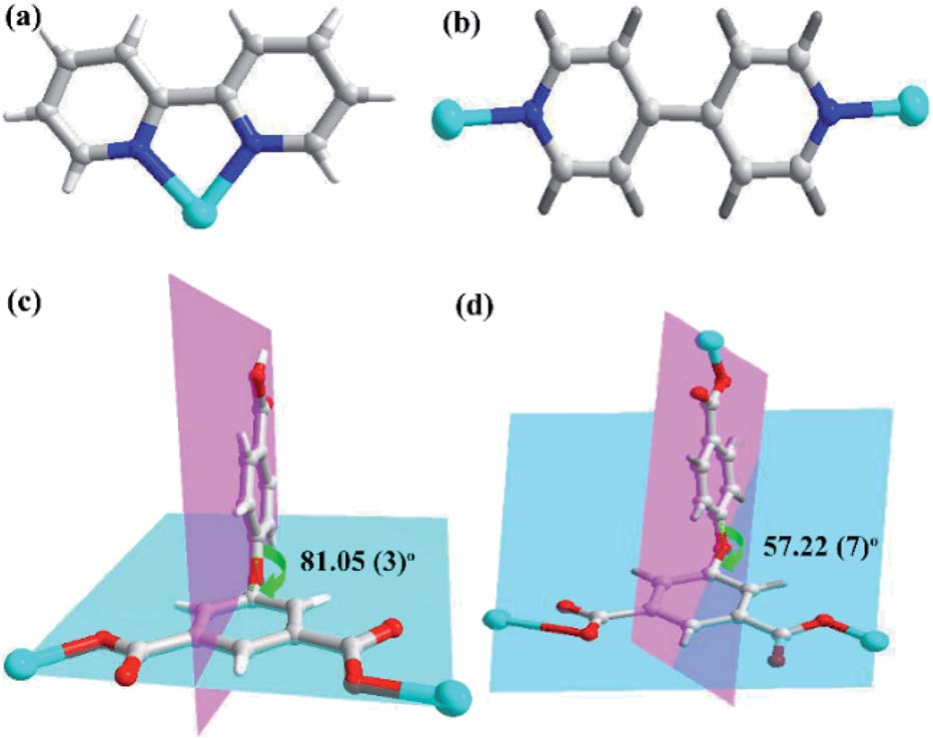
Bonding modes of (a) 2,2′-bpy and (b) 4,4′-bpy ligand with Zn(ii) centers; (c and d) perspective view of the coordination modes of the V-shaped semi-rigid H3L ligand and the dihedral angles between the two phenyl rings of H3L ligands in CPs 1 and 2.

During last few years we are interested in using V-shaped ligands as flexible and hydrophobic bridging unit for the development of varied types of CPs/MOFs for different applications.^11^ In order to gain further new information and also to explore the effect of change in the positions of asymmetrically attached carboxylic groups on the molecular structure and functional properties of CPs/MOFs, in the work presented herein, a symmetric semi-rigid V-shaped multicarboxylate ligands 5-(4′-carboxylphenoxy)isophthalic acid (H_3_L) (Scheme 1) had been selected to obtain two new coordination polymers with formula [Zn(HL)(2,2′-bipy)(H_2_O)]_*n*_ (1) and [Zn_3_(L)_2_(4,4′-bipy)_3_]_*n*_ (2) and (H_3_L = 5-(4′-carboxylphenoxy)isophthalic acid; 4,4′-bipy = 4,4′-bipyridine and 2,2′-bipy = 2,2′-bipyridine). Also, these CPs have been used as photocatalysts for degrading aqueous solution of methyl violet (MV) which indicated that these newly synthesized CPs can be the promising candidate for visible-light-driven photocatalyst for the degradation of organic pollutants. The results of all these investigations are presented herein.

## Experimental

### Materials and method

All the chemicals and reagents used for the syntheses of both the Zn(ii) based CPs were commercially available and used without further purification. The powder X-ray diffraction (PXRD) data was collected using Bruker ADVANCE X-ray diffractometer with Cu-Kα radiation (*λ* = 1.5418 Å) at 50 kV voltage, 20 mA current and scanning rate of 6° min^−1^ with step size of 0.02°. Fourier transform infrared (FT-IR) spectra in KBr discs were recorded using Nicolet Impact 750 FTIR in the range of 400–4000 cm^−1^.

### X-ray crystallography

The single crystal X-ray diffraction data were collected on Bruker SMART APEX diffractometer equipped with graphite monochromated MoKα radiation (*λ* = 0.71073 Å) by using an ω-scan technique. The structure of CPs were solved by direct method (SHLEXS-2014) and refined using the full-matrix least-squares procedure based on *F*^2^ (Shelxl-2014).^12^ All the hydrogen atoms were generated geometrically and refined isotropically using a riding model. All non-hydrogen atoms were refined with anisotropic displacement parameters. The crystallographic details and selected geometrical parameters for both the CPs are enlisted in Tables S1 and S2,† respectively. CCDC number: 1981484 and 1981485.

### Photocatalytic method

The finally divided sample of 1 or 2 (50 mg) was added and dispersed in 50 mL aqueous solution of methyl violet (15 mg L^−1^). The mixture was stirred under dark for 30 min to ensure establishment of adsorption–desorption equilibrium. In other word, after being stirred magnetically in dark to achieve adsorption–desorption equilibrium (*C*_e_ is the adsorption–desorption equilibrium concentration). Thereafter, the photocatalytic degradation of MV was conducted on XPA-7 type photochemical reactor which was equipped with 100 W mercury lamp (mean wavelength 365 nm) with light intensity of 12.7 mW cm^−2^ at quartz tube. During the photocatalytic degradation, aliquots of 5.0 mL were isolated at specific time intervals and separated through centrifugation and then subsequently the characteristic electronic absorption band of MV was recorded using UV-vis spectrophotometer. Additionally, a control experiment was conducted under the similar reaction conditions but without the addition of CPs 1 and 2. The recycle experiments were conducted thrice under the similar reaction conditions. All the data used for analyses are the average value from three times parallel tests.

### Computational details

The possible photocatalytic mechanism with which both the CPs executed the photodegradation of the MV have been explained with the aid of theoretical calculations. The smallest unit of MOF was geometry optimized using the B3LYP functional,^13*a*,13*b*^ using 6-31G** basis set for all the atoms except Zn for which CEP-121G basis set was employed. All the calculations were performed using Gaussian 09 program,^13*c*^ while the density of states and partial density of states for both the compounds were constructed using GaussSum 3.1.^13*d*^

### Synthesis of 1

#### [Zn(HL)(2,2′-bipy)(H_2_O)]_*n*_ (1)

A mixture of H_3_L (0.15 mmol, 0.045 g), 2,2′-bipy (0.25 mmol, 0.048 g) and Zn(NO_3_)_2_·6H_2_O (0.40 mmol, 0.119 g) was taken in 10 mL distilled water and pH value was adjusted to 6.0 with 0.5 mol L^−1^ NaOH aqueous solution. Thereafter, the mixture was transferred in a Teflon-lined stainless steel vessel (25 mL), and heated to 180 °C for 72 h and then cooled to room temperature at a rate of 5 °C h^−1^. Colourless block crystals of 1 were obtained in 66% yield based on zinc. Anal. calcd for 1 (%): C, 55.63; H, 3.36 N, 5.19. Found: C, 55.42; H, 3.39; N, 5.23. IR (KBr, cm^−1^): 3191(v), 2348(w), 1712(v), 1627(m), 1570(m), 1442(s), 1299(m), 1087(m), 973(m), 767(s).

#### [Zn_3_(L)_2_(4,4′-bipy)_3_]_*n*_ (2)

The synthesis procedure of 2 was analogous to that of 1, except that 2,2′-bipy was replaced by 4,4′-bipy (0.25 mmol, 0.048 g). Colorless block crystals of 2 were obtained in 62% yield based on zinc. Anal. calcd for 2 (%): C, 57.05; H, 3.03; N, 6.65. Found: C, 56.87; H, 3.06; N, 6.71. IR (KBr, cm^−1^): 3382(v), 2928(w), 1613(m), 1549(m), 1435(m), 1364(s), 1108(m), 930(s), 774(m), 724(m).

## Results and discussion

### Molecular structure description of [Zn(HL)(2,2′-bipy)(H_2_O)]_*n*_ (1)

**Fig. 1:**
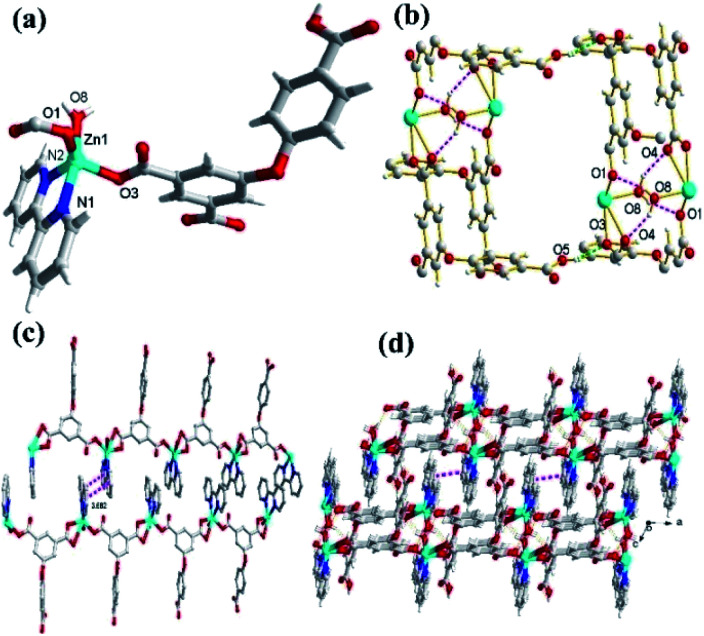
(a) The coordination environment around Zn(ii) ion in 1. (b) Perspective view of 2D network in 1. (c) The 2D supramolecular networks through intermolecular π–π interaction. (d) View of 3D supramolecular frameworks in complex 1.

In the CP 1, the asymmetric unit comprises a Zn1 center, a μ_2_-HL^2−^ linker, one coordinated water and 2,2′-bipy (Fig. 1a). The penta-coordinate Zn1 displays trigonal bipyramid geometry {ZnN_2_O_3_}, where the coordination positions are occupied by two O-donors from two μ_2_-HL^2−^ blocks, one O center from water molecule and a pair of N from 2,2′-bipy donors. The Zn–O bond lengths vary between 2.0287(18)–2.591(4) Å, while Zn–N bond lengths lies between 2.109(2)–2.1134(18) Å. These bonding parameters are in good agreement with previously reported Zn(ii) based analogous compounds.^14^ The ligand HL^2−^ behaves as a μ_2_-linker (Scheme 1) with two COO^−^ groups displaying monodentate-bridging modes while the third COOH functionality remains uncoordinated. In HL^2−^, the dihedral angles between two aromatic rings and a C–O ether–C angle are 81.05(3) and 122.23(4)°, respectively. The HL^2−^ blocks connect the Zn1 centres to generate a 1D zigzag chain structure with the Zn1⋯Zn1 separation of 9.926(8) Å (Fig. 1c). Also, these chains are connected together by the O–H⋯O hydrogen bonds to form a 2D supramolecular network (Fig. 1b). These layers are further packed into a 3D supramolecular network *via* intermolecular π–π interaction (face–face distance amounting to 3.682(4) Å) between 2,2′-bipy ligands of neighbouring layers (Fig. 1c and d).^8,15^

### Molecular structure description of [Zn_3_(L)_2_(4,4′-bipy)_3_]_*n*_ (2)

**Fig. 2:**
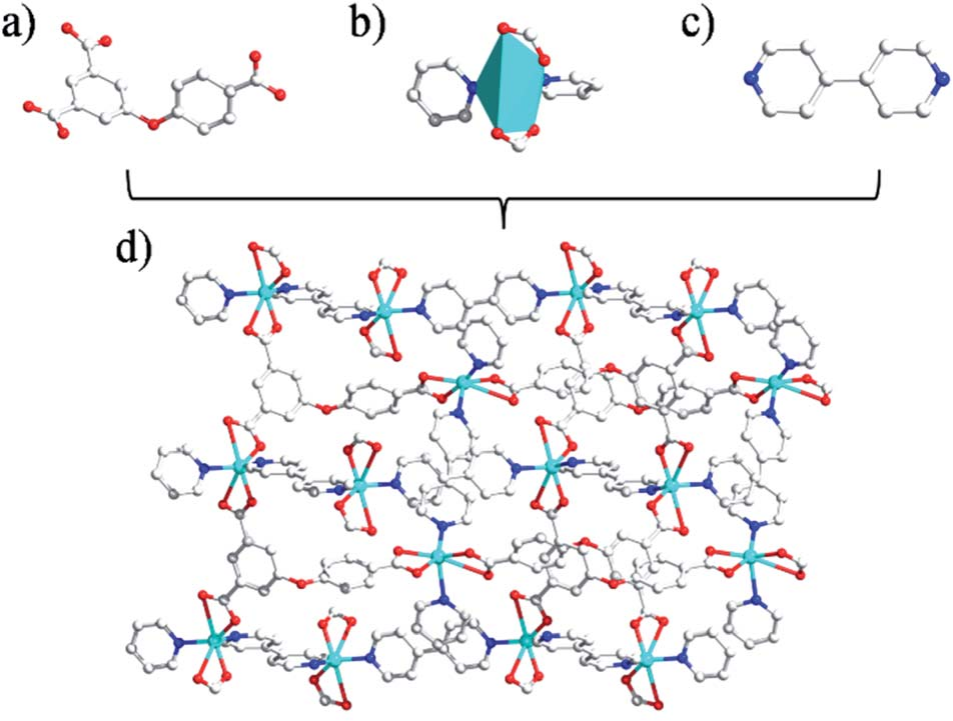
(a and c) The linkers built by coligand; (b) the diagram of coordination geometry for Zn(ii)atoms in 2 (the 30% probability level), all hydrogen atoms and water molecules have been omitted for clarity; (d) 3D network pillared by 4,4′-bpy ligands.

Unlike 1, the CP 2 was obtained by introducing linear N-donor (4,4′-bipy) secondary ligand instead of the chelating N-donor (2,2′-bipy) ligand, which has a complicated 3D frame as expected. In 2, the geometry around the Zn(ii) centers are tetrahedral which are satisfied by two oxygen centers from two L^3−^ ligands in *trans* configuration and two N atoms from 4,4′-bpy ligands (Fig. 2a–c). Unlike 1, in 2 all the H_3_L ligand is completely deprotonated and the L^3−^ moiety acts as a μ_3_-linker (Scheme 1b and d) where all the COO^−^ groups adopt μ-bridging monodentate coordination modes. Besides this, the L^3−^ is considerably bent showing a dihedral angles of 57.22(7)° and 123.46(2)° between two aromatic rings and the C–O ether–C units. The neighbouring zinc subunits are linked by 4-carboxylic groups in *syn*–*anti* coordination mode to form a 1D chain. The adjacent chains are further connected by the L^3−^ ligands to give rise a 2D covalent network. The 4,4′-bipy ligands connect the Zn(ii) ions of neighbouring 2D layered networks as pillar to form a 3D structure (Fig. 2d).^8^ The topological analysis was performed using the concept of the underlying net. After a simplification procedure, an underlying net has been obtained (Fig. S1†). This framework can be defined as a 4-connected net with the point symbol of (4^2^·6^3^·8), by denoting both the Zn(ii) centers as four-connected nodes.

In the presented investigation both the CPs were synthesized by one-pot reaction and afforded different structures and these structures were influenced by the variation in N-donor ligands. The dihedral angles between the two phenyl rings of H_3_L ligands are shown in Scheme 1. In 1, the HL^2−^ ligand connects two adjacent Zn centers to form a single-stranded chain. However, in 2, there are two types of carboxylate chains which were influenced by the flexibility of H_3_L. Meanwhile, the completely deprotonated L^3−^ ligand connects two adjacent Zn centers to generate a layer with different dihedral angles. In other words, in these CPs obtained from mixed ligands, the difference in their molecular structures indicate the significant spacer effect of secondary N-donor ligands which is modulating the molecular structures of coordination polymers as well as the bridging modes and conformations of H_3_L ligand.^8^

### IR, SEM and UV-vis diffuse reflectance spectroscopy (DRS)

In the FTIR spectra, the bands at ∼3400 cm^−1^ arises due to the aqua ligand in 1. The characteristic bands at 1400–1580 cm^−1^ can mainly be attributed to asymmetric and symmetric stretching vibrations of the carboxylato groups. The Δ*ν*[*ν*_as_(COO)–*ν*_s_(COO)] values of 211 and 188 cm^−1^ for 1 and 2, respectively indicates coordination modes of carboxylato groups to the metal centres (Fig. S2†). The band observed at *ca.* 1506 cm^−1^ in both 1 and 2 can be assigned to *ν*(C=N) absorption of the N-donor ligand. In SEM, the morphologies and particle sizes of both the CPs can be viewed. The as-synthesized 1 and 2 consist of collapsed octahedron crystals with a non-uniform diameter *ca.* 700 nm (Fig. S3†). The UV-vis diffuse reflectance spectra of the CPs in the range of 200–800 nm was measured with BaSO_4_ as a reference (Fig. S4†). The absorption spectra revealed that 1 and 2 have an absorption edge around 237 nm. In addition, another absorption band in the region of 250–600 nm for 1 and 2 can be observed. Therefore, 1 and 2 have electronic absorption in the visible range of the spectrum. The band gaps calculated using the Tauc plot were found to be 2.62 eV and 2.93 eV for 1 and 2, respectively which is an important parameter for evaluating light utilization (Fig. S4†).^15*b*^

### Photocatalysis property

**Fig. 3:**
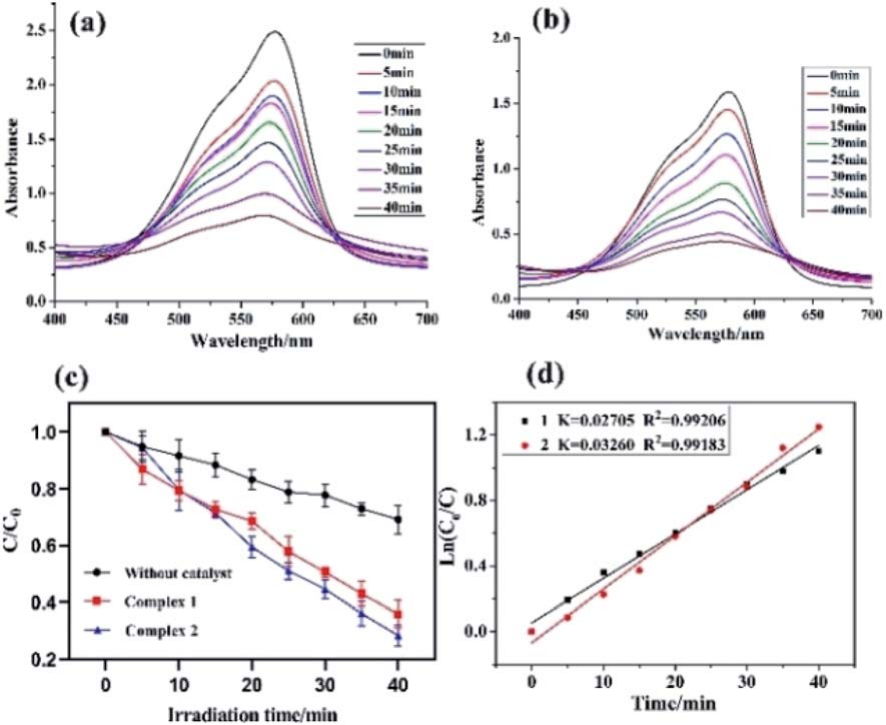
(a and b) the electronic absorption spectra of the MV solutions during the decomposition reaction under UV irradiation in the presence of 1 and 2, respectively; (c) over different catalysts, and the rate constant *k* for degradation of MV; (d) linear-log plot as a function of visible light irradiation time in the presence of 1 and 2.

In this study, both the CPs have been used as photocatalyst for the safe and sustainable photodecomposition of the model aromatic dye methyl violet (MV) a contaminant generally existing in wastewater. The extent of degradation of MV in presence of both the CPs as photocatalysts were monitored by recording the characteristic absorption band of MV at 580 nm (Fig. 3a and b). The variation in the UV-vis absorption intensity related to the concentrations of MV solutions (*C*_*t*_/*C*_0_) against the irradiation time in presence of photocatalysts were also plotted (Fig. 3c). About 64.82% and 78.18% of MV underwent photodegradation after 40 min on UV-vis irradiation in the presence of 1 and 2, respectively (Fig. 3c), while merely 18.66% photodegradation of MV was recorded under the control conditions in the absence of CPs. Therefore, the experiments indicate that CPs 1 and 2 can be used as efficient catalysts for the photocatalytic degradation of MV.

To gain better understanding of the reaction kinetics of MV photodegradation, the experimental data were fitted to the first-order model using Langmuir–Hinshelwood model. The *k* value is the rate constant which had been obtained from the slope and the intercept of the linear plot.^16^ For CPs 1 and 2, the photodegradation rate constants values are 0.02705 min^−1^ and 0.03260 min^−1^, respectively.

The photocatalytic properties of both the CPs reported herein have been compared to some previously reported CPs/MOFs which displayed photocatalytic properties (Table 1)^17^ which suggests that CPs reported herein offered good application as the photocatalyst for the decomposition of the MV and in certain experimental aspects they are advantageous.^17^ In addition to these reports, a MOF-based photocatalyst ZnO@ZIF-8 had been reported which requires more energy by using UV light (300 W high pressure Hg lamp) than the CPs 1 and 2 (100 W high pressure Hg lamp).^18^ Also, the amount of these CPs required during photocatalysis is less than our previously reported photocatalyst (80 mg).^19^ Despite, smaller dosage the CPs 1 and 2 displayed better photodegradation properties under similar condition.^19^

**Table 1:** Performances of some photocatalysts for the degradation of MV

Compounds	Irradiation	Degradation efficiency (%)	Ref.
[Zn_7_(NDC)_5.5_(μ_4_-OH)_3_]	UV	65	17*a*
[Zn_2_(pa)_2_(bip)_2_]	UV	52	17*b*
[Zn_4_(μ_2_-OH)_2_(BDC)_3_(bip)_2_]	UV	82	17*c*
[Zn_2_(fer)_2_]	UV	54	17*d*
[Zn_3_(btc)_2_(bimmb)_2.5_]	UV	92.16	17*e*
Bi_6_O_6_(OH)_2_(NO_3_)	UV	93	17*f*
CuO nanoleaves	UV	96	17*g*
BFO/RGO composites	UV	65.1	17*h*
[Zn(SO_4_)_3_(DMF)_3_]	UV	76	17*i*
Zn(C_40_H_3_6N_4_O_8_)	UV	45	17*j*
[Co(H_2_ODPT)(bpe)(H_2_O)]	UV	32	17*k*
[Zn(tmlb)(bbibp)]_*n*_	UV	97.3	17*l*
1	UV-vis	75	This work
2	UV-vis	65	This work

**Fig. 4:**
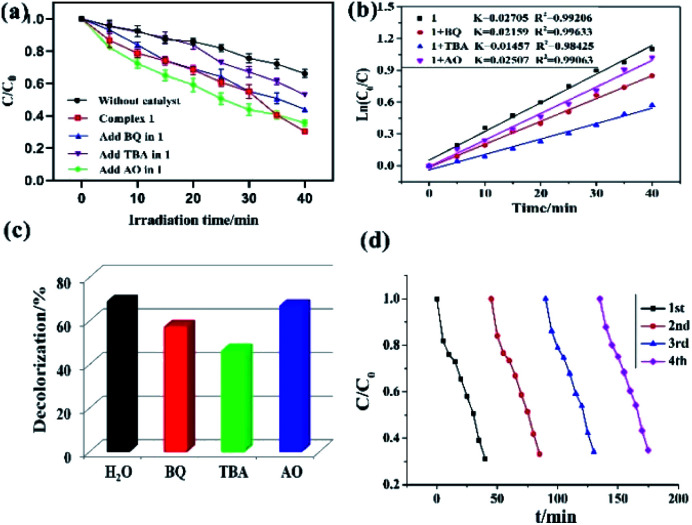
(a) Photocatalytic decomposition of MV solution under UV-vis light irradiation with the use of 1 and different scavenger solutions; (b) linear-log plot as a function of visible light irradiation time in the presence of 1 and different scavengers; (c) photodegradation of the MV solution over 1 in the different scavenger solutions; (d) cycling four runs of the photocatalytic degradation of MV for 1.

**Fig. 5:**
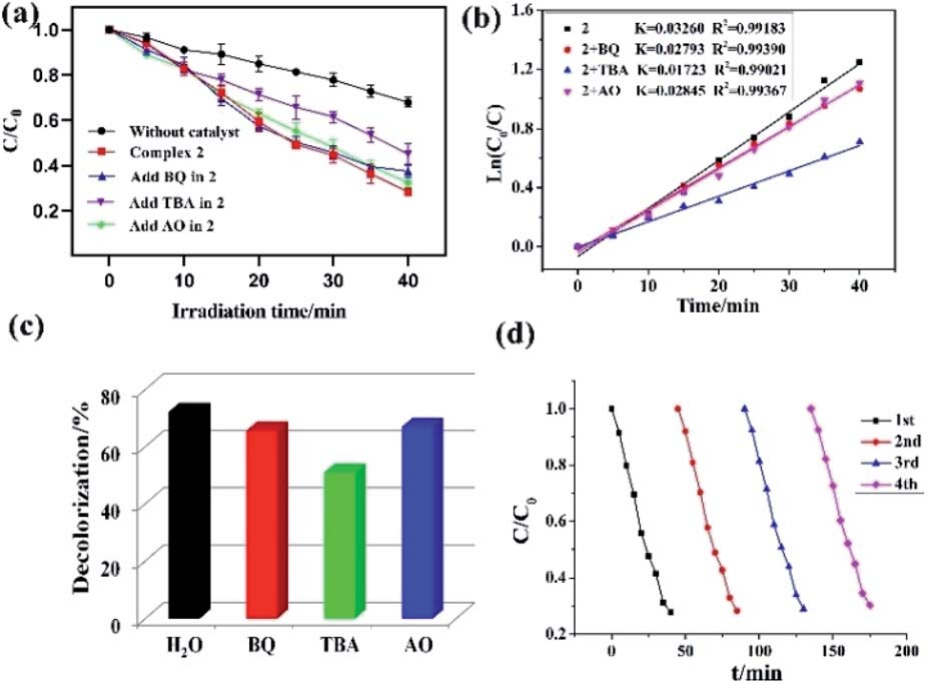
(a) Evolution of the MV concentration under solar irradiation with the different scavenger solutions; (b) linear-log plot as a function of visible light irradiation time in the presence of 2 and different scavengers; (c) photodegradation of the MV solution over 2 in the different scavenger solutions; (d) cycling four runs of the photocatalytic degradation of MV for 2.

To investigate the plausible photocatalytic degradation reaction mechanism and to assess the nature of apposite reactive species which may primarily be responsible for the decomposition of MV,^20–22^ the photodegradation of MV was carried out in the presence of three different quenchers *viz.* tertiary butyl alcohol (TBA) a ·OH radical quencher, benzoquinone (BQ) a O_2_˙^−^ radical quencher and ammonium oxalate (AO) which act as h^+^ radicals quenchers (Fig. 4a and 5a). The photocatalytic reactions executed in presence of these quenchers indicated that TBA dropped the photodegradation of MV in the presence of both the CPs (Fig. 4b, c, 5b and c) (Table 2).

**Table 2:** Parameters of photodegradation reactions of MV in 1 and 2

Materials	MV	
	*k* (min^−1^)	*R* ^2^
1	0.02705	0.99206
1 + BQ	0.02159	0.99633
1 + TBA	0.01457	0.98425
1 + AO	0.02507	0.99063
2	0.03260	0.99183
2 + BQ	0.02793	0.99390
2 + TBA	0.01723	0.99021
2 + AO	0.02845	0.99367

Additionally it is apparent that the rate constants for the decomposition of MV in presence of CPs observed to decrease from 0.02705 to 0.01457 min^−1^ for 1 and 0.03260 to 0.01723 min^−1^ for 2 in the presence of TBA under UV-vis irradiation. Therefore, the photodegradation of MV in presence of ·OH quencher suggest that the photodegradation of MV by the CPs used as photocatalysts is dominated by ·OH radical group.^20^ The exact mechanism in presence of each of these catalysts is challenging and deserves special attention in a future work.^23^ Furthermore, after photocatalysis experiments, the CPs were filtered and characterized using PXRD (Fig. S5 and S6†). The results indicated that CPs isolated after photodegradation experiments displayed nearly identical PXRD pattern as can be obtained for pristine CPs. The PXRD results provides evidence that the CPs are stable enough to be used as photocatalysts to initiate the fresh catalytic cycles. On the basis of this information, the repeated photocatalytic degradation of MV was explored which indicated that the photodegradation rates of MV in presence of 1 and 2 displayed no significant decline even when the photocatalyst was used five times in under similar reaction conditions. Thus, the recycling experiments indicated that both of the CP based materials were stable and can be used as photocatalysts for degradation of MV for atleast four cycles (Fig. 4d and 5d).

**Fig. 6:**
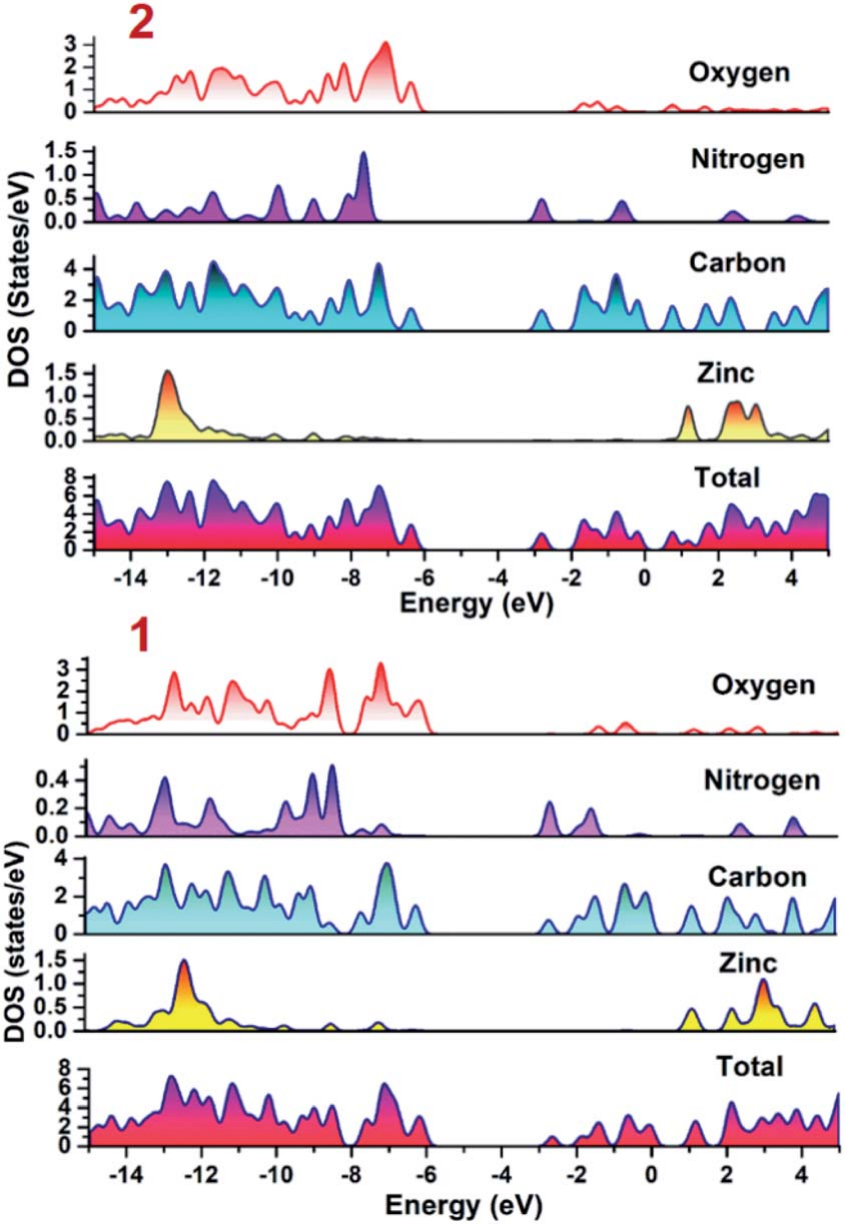
Density of states (DOS) and partials DOS plots for 1 and 2.

To establish the plausible mechanism with the aid of which both the CPs executed photo-degradation of MV, the band structure calculations were performed which was based on density functional theory (*vide supra*). Using DFT calculations, the DOS and partial DOS plots have been constructed (Fig. 6). The plots display that the valence band in both the CPs are having major contributions from aromatic carbons and carboxylate oxygens. Additionally in case of 2 nitrogen centers of 4,4′-bipy ligand is also having contribution in the valence band. However, in both the compounds Zn(ii) centers are offering negligible contribution. Also, the conduction band in both the CPs are having contributions from aromatic carbon and nitrogen centers. Therefore, pDOS plots suggest that in both the CPs electronic transitions are of ligand-to-ligand type. The most probable reason for the observed differences in the photocatalytic performances of CPs may be the band gap differences. Apart from this, in the case of 2, the 4,4′-bipy is acting as a linker and is also contributing in the valence band of the 2 and therefore is not only playing a critical role in formation of framework rather also tuning the electronic communication in 2. This may be also be the another reason for relatively superior photocatalytic performance of 2 in comparison to 1.

Therefore, it can be inferred from the integrated experimental and theoretical calculations that charge transfer operates from valence band (VB) → conduction band (CB) in photo-excited CPs which in turn generates a hole in VB. To revert back to its stable state the CB captures an electron from water molecule which itself gets converted to HO˙ radical which is an active species. The generated HO˙ radicals then decomposes the MV to accomplish the photocatalytic process. The mechanistic pathway through which the entire photocatalysis is operating can be presented as:^11,12^1
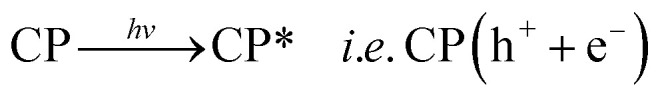

2 CP(h^+^ + e^−^) + H_2_O → CP(e^−^) + HO˙ + H^+^

3 HO˙ + MV → oxidation products → CO_2_ + H_2_O

4 CP(e^−^) + O_2_ → CP(O_2_˙^−^)

5
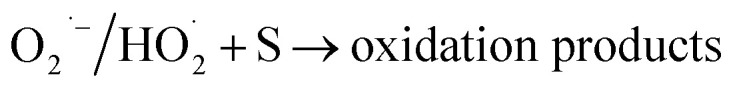


## Conclusion

Two new Zn(ii) based CPs have been synthesized using semi-rigid V-shaped 5-(4′-carboxylphenoxy)isophthalic acid as ligand while 2,2′-bipy and 4,4′-bipy as co-ligands. These newly synthesized CPs possess entirely different topologies and been used as the photocatalysts for the photodecomposition of methyl violet and offered differences in their photocatalytic performances. The variation in photocatalytic performances in these CPs may be arising because of the differences in band gaps as well as the presence of 4,4′-bipy in one of the CP which led to the enhancement in the electron and hole transfer process. In conclusion, the selection of appropriate linker can generate the desirable framework and in turn can fine tune the electron and hole transfer properties in the resulting CPs. These variations in the topologies and electron transfer properties could be utilized to decipher photocatalytic properties in the targeted CPs.

## Conflicts of interest

There are no conflicts to declare.

## Supplementary Material

RA-010-D0RA02222E-s001

RA-010-D0RA02222E-s002
