# Supplementary material for: Structures and photocatalytic properties of two new Zn(ii) coordination polymers based on semi-rigid V-shaped multicarboxylate ligands
Source: RSC Adv. 2020 May 18;10(32):18721–7. doi: 10.1039/d0ra02222e (PMC9053881; doi:10.1039/d0ra02222e)
Supplement: RA-010-D0RA02222E-s001 [file RA-010-D0RA02222E-s001.pdf]

Electronic Supplementary Material (ESI) for RSC Advances.
This journal is © The Royal Society of Chemistry 2020

Supporting Information

Structures and photocatalytic properties of two new Zn(II) coordination polymers based on semi-rigid V-shaped multicarboxylate ligand

Shan-He Zhou[a], Jun Wang[a*], Yi-Wei Liu[b], Yuyu Zhong[b], Yan-Chun Sun[a], Bin Xie[a], Aiqing Ma[b*], Amita Singh,[c] Mohd. Muddassir,[d] Abhinav Kumar[c*]

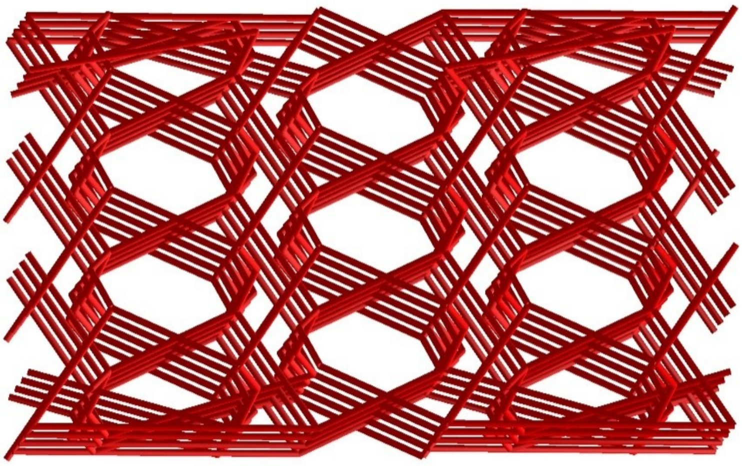

Fig. S1 Topological representation (view along the a axis) of the 4-connected 3D net

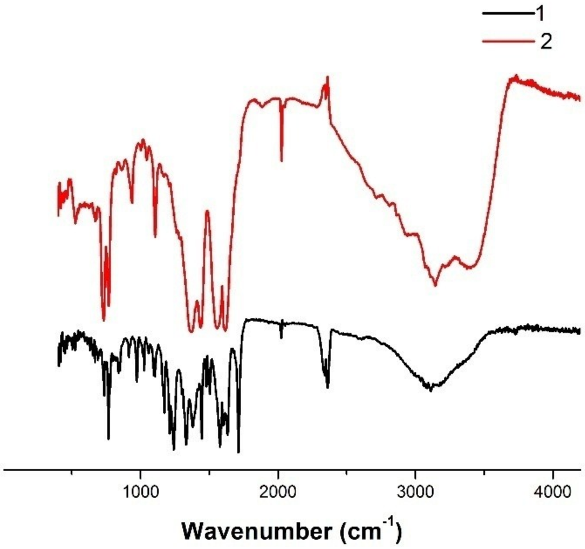

Fig. S2 view of the IR.

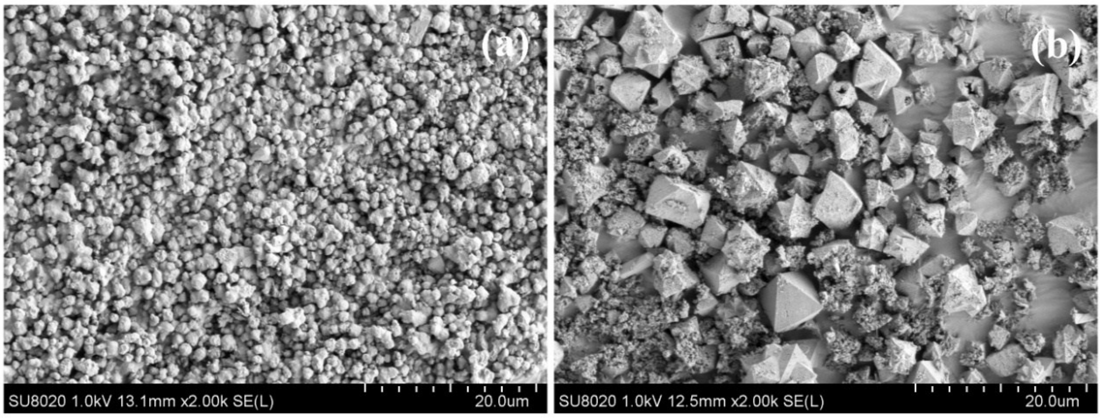

Fig. S3 SEM image of title CP s. The magnifications of picture were 100 thousand.

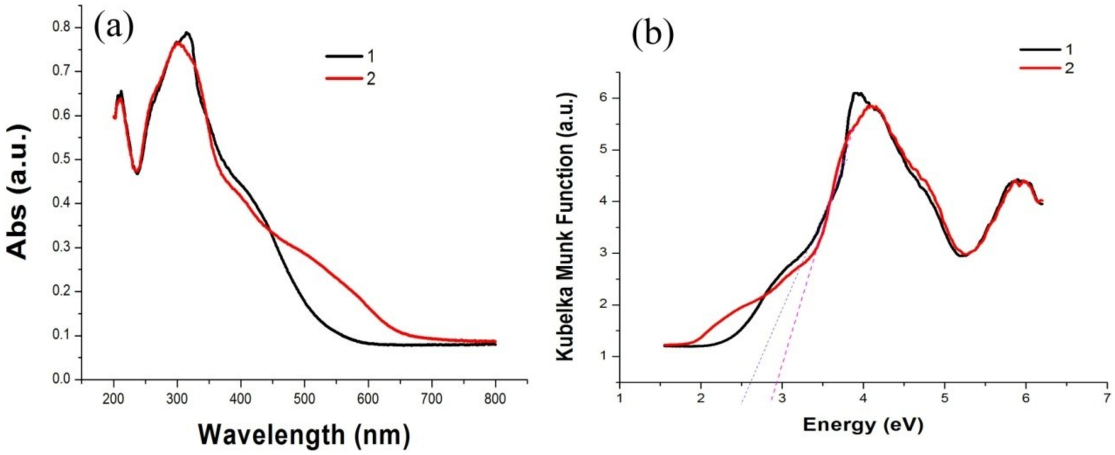

Fig.S4 (a)The UV-visible absorption spectrum of **1** and **2** and (b) Solid-state optical diffuse-reflection spectra of **1** and **2** derived from diffuse reflectance data at ambient temperature.

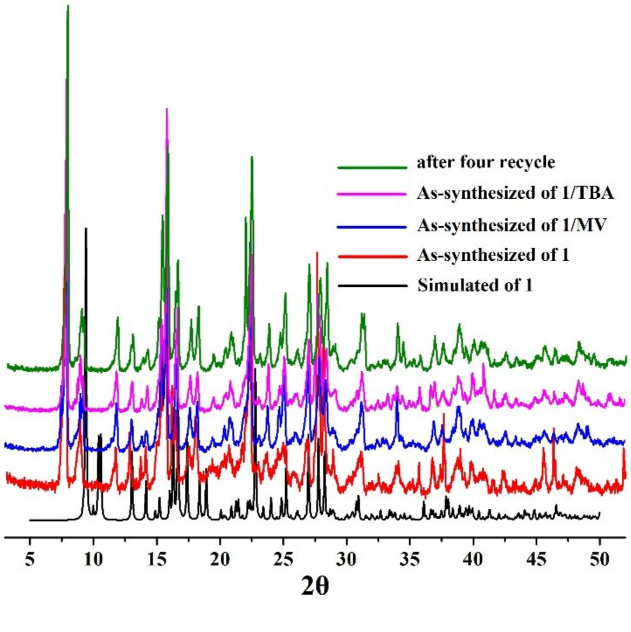

Fig. S5 view of the PXRD patterns of **1** under the different conditions.

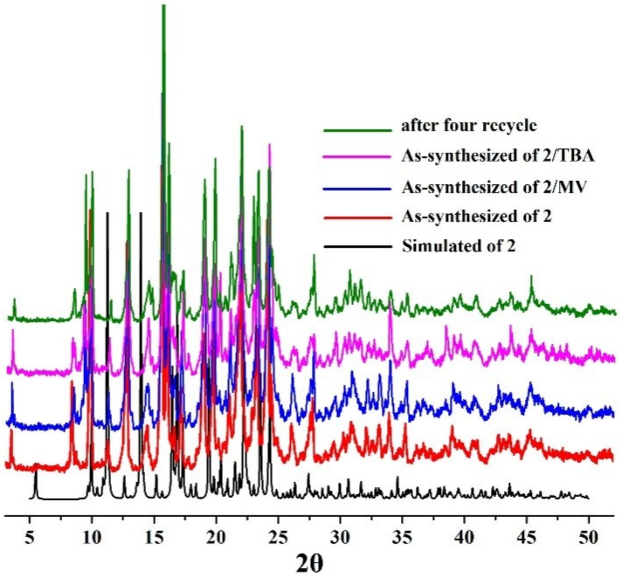

Fig. S6 view of the PXRD patterns of **2** under the different conditions.

Table 1. Crystallographic data and structure refinement details for CPs 1-2

Parameter	**1**	**2**
Formula weight	539.80	1263.07
Crystal system	Triclinic	Monoclinic
Space group	*P-1*	*C2/c*
Crystal Color	Colorless	Colorless
a, Å	9.9215(14)	32.621(5)
b, Å	11.0863(16)	9.236(4)
c, Å	12.2729(17)	18.455(5)
α, °	111.943(2)	90
β, °	102.760(2)	98.21(2)
γ, °	106.552(2)	90
V, Å^3	1116.0(3)	5503(3)
Z	2	4
ρ_{calcd}, g/cm^3	1.606	1.525
μ, mm^{-1}	1.157	2.150
F(000)	552	2568

θ Range, deg	2.4-27.7	4.8-66.0
Reflection Collected	5246	4727
Independent reflections (R_{int})	0.016	0.043
Reflections with $I > 2\sigma(I)$	4233	3321
Number of parameters	332	409
R_1, $wR_2(I> 2\sigma(I))^*$	0.0326, 0.0824	0.0993, 0.2785
R_1, wR_2 (all data)**	0.0417, 0.0866	0.1236, 0.3126

* $R = \sum(F_o - F_c)/\sum(\mathrm{F_o})$, ** $wR_2 = \{\sum[w(F_o^2 - F_c^2)^2]/\sum(F_o^2)^2\}^{1/2}$.

Table 2. Selected bond distances (Å) and angles (deg) for **1-2**

		**1**	
Zn(1)-O(1)	2.0287(18)	Zn(1)-O(8)	2.0951(16)
Zn(1)-N(1)	2.1134(18)	Zn(1)-N(2)	2.109(2)
Zn(1)-O(3)#1	2.0757(18)		
		**2**	
Zn-O(6)	2.229(10)	Zn-N(3)	2.013(12)
Zn-O(6)#1	2.061(11)	Zn-O(7)#1	2.121(7)
Zn(01)-O(1)	1.909(9)	Zn(01)-N(1)	2.015(6)
Zn(01)-N(2)	2.086(8)	Zn(01)-O(4)#2	1.955(6)

		**1**	
O(1)-Zn(1)-O(8)	89.83(7)	O(1)-Zn(1)-N(1)	93.20(7)
O(1)-Zn(1)-N(2)	132.16(7)	O(1)-Zn(1)-O(3)#1	127.92(7)
O(8)-Zn(1)-N(1)	165.01(8)	O(8)-Zn(1)-N(2)	89.53(7)
O(3)#1-Zn(1)-O(8)	94.21(7)	N(1)-Zn(1)-N(2)	77.57(8)
O(3)#1-Zn(1)-N(1)	95.49(7)	O(3)#1-Zn(1)-N(2)	99.82(7)
		**2**	
O(6)-Zn-N(3)	123.9(4)	O(6)-Zn-O(6)#1	86.8(4)
O(1)-Zn-O(7)#1	136.6(6)	O(6)#1-Zn-N(3)	91.3(5)
O(7)#1-Zn-N(3)	88.6(5)	O(6)#1-Zn-O(7)#1	62.3(5)
O(1) -Zn(01)-N(1)	139.6(3)	O(1)-Zn(01)-N(2)	101.7(3)
O(1)-Zn(01)-O(4)#2	106.0(3)	N(1)-Zn(01)-N(2)	100.3(3)
O(4)#2-Zn(01)-N(1)	106.3(2)	O(4)#2-Zn(01)-N(2)	93.0(3)

Symmetry Cddes: **For 1**: #1= 1+x, y, z. **For 2**: #1 = 1-x, y, 3/2 -z; #2 = x, 1-y, -1/2+z.
